# The mean diffusivity of forceps minor is useful to distinguish amnestic mild cognitive impairment from mild cognitive impairment caused by cerebral small vessel disease

**DOI:** 10.3389/fnhum.2022.1010076

**Published:** 2022-11-09

**Authors:** Yue Zhang, Lu Lin, Mengmeng Feng, LingYan Dong, Yiren Qin, Huan Su, Zheping Zhou, Hui Dai, Yueju Wang

**Affiliations:** ^1^Phase I Clinical Trial Center, Chongqing University Cancer Hospital, Chongqing, China; ^2^School of Nursing, Medical College of Soochow University, Suzhou, China; ^3^Department of Radiology, The First Affiliated Hospital of Soochow University, Suzhou, China; ^4^Department of Gerontology, The First Affiliated Hospital of Soochow University, Suzhou, China; ^5^Department of Neurology, The First Affiliated Hospital of Soochow University, Suzhou, China

**Keywords:** cerebral small vessel disease, diffusion tensor imaging, neuropsychological tests, ROI-based analyses, amnestic mild cognitive impairment

## Abstract

**Objectives:**

In recent years, the desire to make a more fine-grained identification on mild cognitive impairment (MCI) has become apparent, the etiological diagnosis of MCI in particular. Nevertheless, new methods for the etiological diagnosis of MCI are currently insufficient. The objective of this study was to establish discriminative measures for amnestic mild cognitive impairment (a-MCI) and MCI caused by cerebral small vessel disease (CSVD).

**Materials and methods:**

In total, 20 normal controls (NCs), 33 a-MCI patients, and 25 CSVD-MCI patients performed comprehensive neuropsychological assessments concerning global cognitive function and five cognitive domains as well as magnetic resonance imaging scan with diffusion tensor imaging (DTI). Diffusion parameters including fractional anisotropy and mean diffusivity of 20 major white matter metrics were obtained by ROI-based analyses. The neuropsychological tests and diffusion measurements were compared and binary logistic regression was used to identify the best differential indicator for the two MCI subgroups. The discriminating power was calculated by receiver operating characteristic analysis.

**Results:**

Amnestic mild cognitive impairment group showed significant impairment in memory and language function, while CSVD-MCI group revealed more deficits in multi-cognitive domains of memory, language, attention and executive function than controls. Compared to the a-MCI, CSVD-MCI was significantly dysfunctional in the executive function. The CSVD-MCI group had decreased fractional anisotropy and increased mean diffusivity values throughout widespread white matter areas. CSVD-MCI presented more severe damage in the anterior thalamic radiation, forceps major, forceps minor and right inferior longitudinal fasciculus compared with a-MCI group. No significant neuropsychological tests were found in the binary logistic regression model, yet the DTI markers showed a higher discriminative power than the neuropsychological tests. The Stroop test errors had moderate potential (AUC = 0.747; sensitivity = 76.0%; specificity = 63.6%; *P* = 0.001; 95% CI: 0.617–0.877), and the mean diffusivity value of forceps minor demonstrated the highest predictive power to discriminate each MCI subtype (AUC = 0.815; sensitivity = 88.0%; specificity = 72.7%; *P* < 0.001; 95% CI: 0.698–0.932).

**Conclusion:**

The mean diffusivity of forceps minor may serve as an optimal indicator to differentiate between a-MCI and CSVD-MCI.

## Introduction

Mild cognitive impairment (MCI) is a heterogeneous cognitive impairment syndrome and a precursor of various subtypes of dementia (Anderson, 2019), such as Alzheimer’s disease (AD), vascular dementia, dementia with Lewy bodies, frontotemporal dementia, and so on. MCI patients show mild memory loss or other cognitive impairment, with basically preserved activities of daily living (Petersen and Morris, 2005). Statistics showed that 40% of MCI patients eventually develop dementia (Mitchell and Shiri-Feshki, 2009). It is particularly challenging for clinicians to identify individual MCI patients which transit from the symptomatic pre-dementia phase to dementia onset. Even more challenging is the etiological diagnosis of MCI, which is essential for the early treatment of different MCI subtypes.

We focused on two types of MCI, one is amnestic mild cognitive impairment (a-MCI), another is MCI caused by cerebral small vessel disease (CSVD-MCI). Patients with a-MCI are at higher risks of evolving toward AD. Traditionally, a-MCI is considered a typical prodromal stage of AD (Petersen, 2004; Gómez-Soria et al., 2021). With the intensive study of a-MCI, researchers have proposed the concept of the clinical subtype of a-MCI, including a-MCI-single domain (a-MCI-s) and a-MCI-multiple domain (a-MCI-m) (Petersen, 2004). Actually, identifying a-MCI-m patients by neuropsychological testing is challenging given that the deficits of multiple cognition are the defining feature of CSVD-MCI and commonly characterize a-MCI-m. There is no uniform standard for the etiological diagnosis of a-MCI and CSVD-MCI.

The cognitive profile of AD and CSVD has been extensively studied over the last decade with an emerging consensus regarding the profile which typifies two disease. Study reported that the neuropsychological profile in CSVD generally showed more deficits in memory, executive function, attention, and visual-spatial skills, whereas AD showed poor performance in memory tests (Graham et al., 2004). However, studies on the differences between the two MCI subtypes have been lacking, and previous studies have reported controversial findings on damage of cognitive domains in the two MCI patients. A study directly compared the cognition of MCI patients due to AD with that of CSVD-MCI patients discovered that the former performed relatively poorly on memory tests but performed better on executive function (Zhou and Jia, 2009). Nevertheless, some studies have failed to identify cognitive differences among patients with different MCI subtypes and the findings are heterogeneous (Meyer et al., 2002; Loewenstein et al., 2006). It was not clear whether the degree of impairment in various cognitive domains of the two MCI subtypes were different, and what role neuropsychological tests play in etiological diagnosis of MCI. We still need to further explore the differences between a-MCI and CSVD-MCI patients by means of neuropsychological tests. The diagnosis of MCI and the assessment of the degree of cognitive decline still rely mainly on cognitive testing of which the ability to diagnose the etiology of MCI is insufficient. We also need more objective tools to distinguish between the MCI subtypes.

White matter (WM) involvement in CSVD and its early stage has been demonstrated by many neuroimaging studies (Wardlaw et al., 2013; Benveniste and Nedergaard, 2022). Many studies have confirmed that white matter lesions (WMLs) can also be observed in the early stage of AD, and even WM impairment precedes gray matter changes (Li et al., 2015; Sindi et al., 2015). Changes of brain pathology in AD are usually years or even decades before clinical symptoms, and changes of WM microstructure exist before cognitive impairment (Amieva et al., 2008; Sperling et al., 2013). These neuroimaging findings provide new research insights and approaches for differentiating the two MCI subtypes. However, it is unclear whether there are different WM microstructural abnormalities between a-MCI and CSVD-MCI. It is necessary to differential diagnose a-MCI and CSVD-MCI based on objective imaging characteristics not only for the in-depth understanding of degenerative neural changes of MCI but also for the different treatment and conversion judgment for the two subtypes.

Diffusion tensor imaging (DTI) is the most sensitive magnetic resonance technique for detecting the integrity of WM tracts in the early stages of dementia by describing the diffusion motion of water molecules in three-dimensional tissues (Preti et al., 2012). The most common DTI metrics include fractional anisotropy (FA) and mean diffusivity (MD). FA reflects the directionality of molecular diffusion, and MD reveals the average displacement of water molecules (Chua et al., 2008). Decreased FA and increased MD values reflect abnormalities in the WM microstructure. By applying DTI indices, we were able to assess the integrity of WM tracts more accurately and thoroughly in this study.

In widespread WM areas, abnormal DTI indices have been found in patients with AD as well as patients with MCI (Chua et al., 2008; Nishioka et al., 2015). Furthermore, researchers found that WM abnormalities were associated with various cognitive dysfunctions (Delbeuck et al., 2007; Fellgiebel et al., 2008; Mascalchi et al., 2019). However, knowledge of the difference between WM tracts microstructural alterations and cognitive performance in patients with a-MCI and CSVD-MCI remains limited. We hope to provide new insights into the anatomical correlates of the two etiologically different MCI subtypes using neuropsychological assessments and DTI analysis with ROI-based analyses, and then provide a reliable alternative to differentiate between them.

The present study aimed to:(a) explore the cognitive profiles and WM tracts abnormalities of each MCI subtype; (b) establish discriminative measures for these two MCI patients.

## Materials and methods

### Participants

A total of 78 participants matched by age and education level were enrolled in this study: 33 a-MCI patients (12 males and 21 females, with average age 72.33 ± 9.400 years), 25 CSVD-MCI patients (15 males and 10 females, with average age 74.44 ± 7.995 years), and 20 normal controls (NCs) (8 males and 12 females, with average age 69.85 ± 9.326 years). This study was approved by the Institutional Review Board of the First Affiliated Hospital of Soochow University and informed consents were obtained from all participants. All participants performed a series of neuropsychological assessments and 3.0-T magnetic resonance imaging (MRI) scan. The diagnostic criteria for MCI in this study was in accordance with the criteria of Petersen (2004): (a) complaint for cognitive decline; (b) smaller than –1.5 standard deviation from mean of local population in at least one cognitive domain with the standard neuropsychological assessments; (c) preserved basic activities of daily living (BADL) with slightly impaired instrumental activities of daily living (IADL) [as measured by Activities of Daily Living Scale (ADL)]; (d) failure to meet the Diagnostic and Statistical Manual of Mental Disorders, Fourth Edition criteria for dementia (DSM-IV); (e) 18 ≤ the Montreal Cognitive Assessment (MoCA) < 26 adjusted for age and education (Nasreddine et al., 2005).

### Inclusion criteria for amnestic mild cognitive impairment group

(a) MCI was diagnosed using the above criteria; (b) subjective memory complaint and an objective memory impairment for age (Petersen, 2004); (c) medial temporal lobe atrophy (MTA) visual scale (Scheltens and van de Pol, 2012) score ≥ 2 and Fazekas scale (Fazekas et al., 1987) scores ≤ 2.

### Inclusion criteria for cerebral small vessel disease-mild cognitive impairment group

(a) Meeting the above diagnostic criteria of MCI; (b) MTA scale score ≤ 1 and Fazekas scale score ≥ 3.

### Inclusion criteria for normal control group

(a) Without subjective cognitive decline; (b) no objective cognitive impairment determined by neuropsychological assessments, namely, the Mini-mental State Examination (MMSE) ≥ 27 and MoCA ≥ 26; (c) no abnormalities in MRI; (d) normal ADL.

### Exclusion criteria

Participants in the three groups were excluded if they exhibited any of the following conditions:(a) aged under 60 years old; (b) having a history of stroke, brain tumors, epilepsy, or other neurological diseases that may lead to cognitive impairment; (c) having severe depression, schizophrenia and other mental disorders; (d) having other serious physical diseases or contraindications for MRI; (e) having visual or auditory abnormalities that make neuropsychological assessments infeasible; (f) having insufficient Mandarin language abilities to complete the assessments; (g) having signs of large vascular diseases, such as cortical, and/or cortico-subcortical, or non-lacunar infarcts and watershed infarcts or hemorrhages; (h) image artifacts caused by head movement during MRI.

### Neuropsychological assessments

The cognitive function of all the subjects was evaluated by an experienced neuropsychologist. MMSE and the Beijing version of MoCA (Lu et al., 2011) were used to assess global cognitive function. To evaluate memory, language, attention, executive, and visuospatial function, we carried out a battery of neuropsychological tests including the Rey auditory verbal learning test (RAVLT); verbal fluency task (VFT); digital span test (DST); Stroop color word test (SCWT); clock drawing test (CDT; four points). The ADL scale was used to assess activities of daily living while the Hamilton Depression Rating Scale (HAMD) and Hamilton Anxiety Rating Scale (HAMA) were used for mental status examination.

### Magnetic resonance imaging acquisition

All subjects underwent a cranial MRI scan by an experienced imaging physician using the same 3.0-T scanner (Signa HDxt, GE Healthcare, Milwaukee, WI, USA) with an eight-channel head coil. Single spin echo diffusion-weighted echo planar imaging (EPI) sequence was used to obtain DTI data through the following parameters: repetition time (TR) 17000 ms, echo time (TE) 85.4 ms, flip angle 90°, field of view (FOV) 256 × 256 mm, matrix size 128 × 128, voxel size 2 mm × 2 mm × 2 mm, slice thickness 2 mm, 30 different diffusion gradient directions, *b* = 1000 s/mm^2^, 5 non-diffusion weighted *b* = 0 s/mm^2^. Meanwhile, T1-weighted images were collected with scanning parameters as follows: TR 6.50 ms, TE 2.80 ms, inversion time (TI) 900 ms, flip angle 8°, FOV 256 × 256 mm, number of slices 176, and slice thickness 1 mm. Axial T2-weighted and FLAIR sequences were obtained to assess white matter hyperintensity (WMH) and hippocampal atrophy.

### Diffusion tensor imaging data processing

All DTI data were processed with PANDA (Cui et al., 2013) toolbox based on FMRIB Software Library v5.0 involving several steps (brain extraction, DTI images format conversion, realignment, eddy current and motion artifact correction, FA calculation, and diffusion tensor tractography). When tracking WM fibers, a FA value threshold was set at 0.2, and a turning angle threshold of the Fiber Assignment by Continuous Tracking (FACT) algorithm was set at 45° (Basser et al., 2000). The FA and MD standard templates in the Montreal Neurological Institute (MNI) space were non-linearly registered to the FA and MD images in the native space using FSL’s FNIRT command to obtain a conversion matrix, which was used to align the 20 ROIs of the JHU WM Tractography Atlas (Hua et al., 2008) template with the individual space of each subject. Each subject produced 20 WM tracts, and then the FA and MD values of each WM tract were calculated. The main 20 WM tracts were selected as follows: anterior thalamic radiation (ATR); corticospinal tract (CST); cingulum (cingulate gyrus; CgC); cingulum (hippocampus; CgH); forceps major (Fma); forceps minor (Fmi); inferior fronto-occipital fasciculus (IFO); inferior longitudinal fasciculus (ILF); superior longitudinal fasciculus (SLF); uncinate fasciculus (UF); superior longitudinal fasciculus-temporal part (tSLF). All of the tracts were evaluated in both hemispheres, except for the Fma and Fmi.

### Visual assessments of white matter hyperintensity and hippocampal atrophy

One experienced imaging physician used two visual rating scales to identify WMH and hippocampal atrophy on baseline images in each subject without knowing the clinical information of the subjects. Fazekas scale was used for quantification of WMH, MTA visual scale was used to measure hippocampal atrophy, and higher hemispheric scores were used as the MTA scores of the subject.

### Statistical analysis

Statistical analyses were performed using the Statistical Package for Social Science software (SPSS, Version 20.0). The categorical demographic variables were presented in terms of frequency and percentage (%), and group comparisons were made using Pearson χ^2^ two-tailed test, with continuity correction for *n* < 5. Continuous demographic variables were presented as mean ± standard deviation, or the median and the interquartile range. One-way analyses of variance (ANOVA) and non-parametric tests were used to compare the differences across groups. The neuropsychological scores and the DTI parameters of 20 WM tracts among three groups were examined by analysis of covariance (ANCOVA) with age, gender, and education as covariates. Bonferroni correction was conducted to adjust the false-positive rate (*P* < 0.05/20), and significant results further underwent pairwise comparison. In addition, the neuropsychological tests and the tracts with significant between-group differences were further included as covariates in the binary logistic regression model, using the forward method, while the MCI subtype was used as dependent variable, and age, gender, and years of education as controlled variables. Finally, the discriminant validity of independent variables were explored by receiver operating characteristic (ROC) analysis. The significant threshold was set at *P* < 0.05.

## Results

### Clinical data, neuropsychological scores, and visual scores

As shown in Table 1, there were no differences across groups in age, gender, education level, and the history of diabetes mellitus, hyperlipemia, smoking, and drinking. However, there were group differences in the history of hypertension. The proportion of participants with hypertension in the CSVD-MCI group was significantly higher than that in the NC and a-MCI groups (76.0, 35.0, and 33.3%). Compared with the NC group, the a-MCI group showed significantly lower scores in MMSE, MoCA, RAVLT-IR, RAVLT-DR, and VFT, indicating that a-MCI showed significant impairment in memory and language function. As shown in Table 1, there were significant differences in MMSE, MoCA, RAVLT-IR, RAVLT-DR, VFT, DST, SCWT A performance time, SCWT B performance time, SCWT C performance time, SCWT A errors, and SCWT C errors between the NC and CSVD-MCI groups, demonstrating that CSVD-MCI had deficits in multi-cognitive domains of memory, language, attention, and executive function. A statistically significant difference between the a-MCI and CSVD-MCI subjects was only present in the scores of SCWT C performance time and errors, reflecting that the a-MCI group had better performance in executive function than the CSVD-MCI group. HAMD, HAMA, ADL scores among the three groups were not statistically different.

**TABLE 1:** Group comparisons of clinical data, neuropsychological scores, and visual scores.

Variables	NC (*n* = 20)	a-MCI (*n* = 33)	CSVD-MCI (*n* = 25)	χ^2^/*F*-value	*P*-value
Age (years)	69.85 ± 9.326	72.33 ± 9.400	74.44 ± 7.995	1.460	0.239
Male/Female	8/12	12/21	15/10	3.471	0.176
Education (years)	11.10 ± 3.478	8.42 ± 5.238	9.80 ± 4.891	2.039	0.137
Hypertension	7 (35.0%)	11 (33.3%)	19 (76.0%)	6.247	0.002^†‡^
Diabetes mellitus	1 (5.0%)	8 (24.2%)	4 (16.0%)	3.197	0.182
Hyperlipemia	2 (10.0%)	6 (18.2%)	3 (12.0%)	0.746	0.771
Smoking	2 (10.0%)	2 (6.1%)	1 (4.0%)	0.678	0.712
Drinking	1 (5.0%)	2 (6.1%)	1 (4.0%)	0.390	1.000
MMSE	28.10 ± 1.334	23.91 ± 3.565	25.16 ± 2.853	10.750	<0.001^ab^
MoCA	27.05 ± 1.905	21.67 ± 2.217	22.84 ± 2.734	29.363	<0.001^ab^
RAVLT-IR	43.90 ± 10.203	27.79 ± 9.178	29.48 ± 9.328	14.602	<0.001^ab^
RAVLT-DR	9.45 ± 3.591	3.82 ± 3.157	4.48 ± 3.164	17.685	<0.001^ab^
VFT	20.60 ± 3.394	14.64 ± 4.091	14.36 ± 3.616	16.373	<0.001^ab^
DST	13.60 ± 1.930	12.33 ± 1.915	11.76 ± 1.562	3.527	0.035^b^
CDT	3.80 ± 0.616	3.33 ± 0.854	3.24 ± 0.926	1.894	0.158
SCWT A (s)	23.16 ± 6.382	30.65 ± 9.064	34.75 ± 11.626	5.568	0.006^b^
SCWT B (s)	19.60 ± 5.474	24.70 ± 8.355	27.47 ± 9.079	3.524	0.035^b^
SCWT C (s)	32.67 ± 9.361	40.86 ± 14.685	54.94 ± 29.742	6.075	0.004^bc^
SCWT A errors	0 (0, 0)	0 (0, 0.5)	0 (0, 2.5)	6.923	0.031^b^
SCWT B errors	0 (0, 0)	0 (0, 0)	0 (0, 1)	1.057	0.589
SCWT C errors	0 (0, 1.75)	1 (0, 3)	4 (1.5, 8.5)	14.598	0.001^bc^
HAMD	0 (0, 3)	1 (0, 4)	0 (0, 4.5)	1.166	0.558
HAMA	0 (0, 3)	1 (0, 2.5)	0 (0, 3.5)	0.304	0.859
ADL	20.70 ± 2.364	22.67 ± 5.661	22.64 ± 7.158	0.971	0.384
Fazekas scores	0 (0, 1)	0 (0, 1)	3 (1.25, 4)	40.824	<0.001^bc^
MTA scores	1 (0.25, 1.75)	2 (1, 3)	1 (0, 2)	10.835	0.002^ac^

NC, normal controls; a-MCI, amnestic mild cognitive impairment; CSVD-MCI, mild cognitive impairment resulting from cerebral small vessel disease; MMSE, mini-mental state examination; MoCA, Montreal cognitive assessment; RAVLT-IR, Rey auditory verbal learning test immediate recall; RAVLT-DR, Rey auditory verbal learning test delayed recall; VFT, verbal fluency test; DST, digital span test; CDT, clock drawing test; SCWT, Stroop color word test; HAMD, Hamilton depression rating scale; HAMA, Hamilton anxiety rating scale; ADL, activities of daily living; MTA, medial temporal lobe atrophy.

*Post-hoc* paired comparisons showed significant group differences: ^†^*P* < 0.05/3 between NC and CSVD-MCI; ^‡^*P* < 0.05/3 between a-MCI and CSVD-MCI; ^a^*P* < 0.05 between NC and a-MCI; ^b^*P* < 0.05 between NC and CSVD-MCI; ^c^*P* < 0.05 between a-MCI and CSVD-MCI.

### Fractional anisotropy values

**FIGURE 1:**
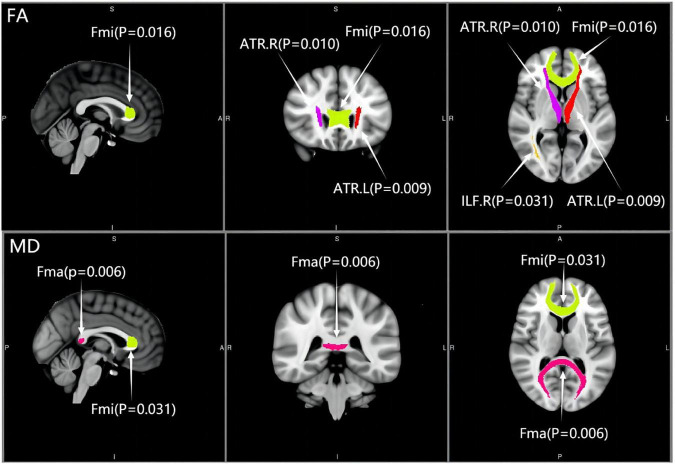
Distribution of significantly differential white matter tracts across the two mild cognitive impairment groups. ATR, anterior thalamic radiation; Fma, forceps major; Fmi, forceps minor; ILF, inferior longitudinal fasciculus; L, left; R, right; FA, fractional anisotropy; MD, mean diffusivity.

Compared with the controls, except for CST and tSLF, the FA values of other WM tracts showed significant decrease in both MCI groups. The results of post-analysis showed that the FA of CgC, CgH, Fma, Fmi, left IFO, ILF, SLF, and UF in the a-MCI and NC groups were significantly different. CSVD-MCI group showed significantly reduced FA values widespread in most of the WM regions except for the bilateral CST, right CgC, and bilateral tSLF. Moreover, compared with the CSVD-MCI group, the a-MCI group presented significantly higher FA values in the bilateral ATR, Fmi and right ILF, as shown in Tables 2, 3 and Figure 1.

**TABLE 2:** Comparisons of fractional anisotropy (FA) values.

Tracts	NC (*n* = 20)	a-MCI (*n* = 33)	CSVD-MCI (*n* = 25)	*F*-value	*P*-value
ATR.L	0.322 ± 0.042	0.317 ± 0.035	0.286 ± 0.033	5.400	0.007^bc^
ATR.R	0.321 ± 0.049	0.304 ± 0.036	0.271 ± 0.033	7.008	0.002^bc^
CST.L	0.486 ± 0.047	0.482 ± 0.032	0.460 ± 0.037	2.089	0.131
CST.R	0.489 ± 0.043	0.487 ± 0.033	0.464 ± 0.034	2.725	0.072
CgC.L	0.409 ± 0.048	0.352 ± 0.049	0.355 ± 0.053	6.746	0.002^ab^
CgC.R	0.385 ± 0.075	0.333 ± 0.040	0.357 ± 0.058	4.053	0.021^a^
CgH.L	0.325 ± 0.043	0.270 ± 0.044	0.280 ± 0.046	9.269	<0.001^ab^
CgH.R	0.322 ± 0.071	0.259 ± 0.030	0.266 ± 0.061	7.451	0.001^ab^
Fma	0.495 ± 0.034	0.462 ± 0.048	0.434 ± 0.045	8.352	0.001^ab^
Fmi	0.387 ± 0.041	0.359 ± 0.034	0.331 ± 0.029	13.841	<0.001^abc^
IFO.L	0.386 ± 0.047	0.353 ± 0.033	0.344 ± 0.026	6.765	0.002^ab^
IFO.R	0.383 ± 0.066	0.364 ± 0.036	0.337 ± 0.029	4.288	0.017^b^
ILF.L	0.402 ± 0.065	0.363 ± 0.032	0.339 ± 0.024	9.834	<0.001^ab^
ILF.R	0.405 ± 0.063	0.367 ± 0.032	0.336 ± 0.022	12.711	<0.001^abc^
SLF.L	0.336 ± 0.044	0.307 ± 0.032	0.297 ± 0.025	5.325	0.007^ab^
SLF.R	0.337 ± 0.039	0.307 ± 0.033	0.301 ± 0.033	5.625	0.005^ab^
UF.L	0.345 ± 0.041	0.308 ± 0.045	0.317 ± 0.031	5.890	0.004^ab^
UF.R	0.346 ± 0.044	0.310 ± 0.048	0.314 ± 0.035	4.929	0.010^ab^
tSLF.L	0.400 ± 0.055	0.398 ± 0.034	0.371 ± 0.049	2.460	0.093
tSLF.R	0.430 ± 0.038	0.402 ± 0.065	0.383 ± 0.049	2.079	0.132

NC, normal controls; a-MCI, amnestic mild cognitive impairment; CSVD-MCI, mild cognitive impairment resulting from cerebral small vessel disease; ATR, anterior thalamic radiation; CST, corticospinal tract; CgC, cingulum (cingulate gyrus); CgH, cingulum (hippocampus); Fma, forceps major; Fmi, forceps minor; IFO, inferior fronto-occipital fasciculus; ILF, inferior longitudinal fasciculus; SLF, superior longitudinal fasciculus; UF, uncinate fasciculus; tSLF, superior longitudinal fasciculus-temporal part; L, left; R, right; FA, fractional anisotropy. FA values are mean ± SD.

*Post-hoc* paired comparisons showed significant group differences: ^a^*P* < 0.05 between NC and a-MCI; ^b^*P* < 0.05 between NC and CSVD-MCI; ^c^*P* < 0.05 between a-MCI and CSVD-MCI.

**TABLE 3:** Details in the *post-hoc* analysis of significant tracts across three groups.

Tracts	ANCOVA	*Post-hoc* analysis			Effect size
	*P*-value	NC vs. a-MCI	NC vs. CSVD-MCI	a-MCI vs. CSVD-MCI	Partial eta square
ATR.L.FA	0.007	1.000	0.037	0.009	0.130
ATR.R.FA	0.002	1.000	0.003	0.010	0.163
Fmi.FA	<0.001	0.012	<0.001	0.016	0.278
ILF.R.FA	<0.001	0.012	<0.001	0.031	0.262
Fma.MD	<0.001	0.032	<0.001	0.006	0.266
Fmi.MD	<0.001	0.030	<0.001	0.031	0.239

NC, normal controls; a-MCI, amnestic mild cognitive impairment; CSVD-MCI, mild cognitive impairment resulting from cerebral small vessel disease; ATR, anterior thalamic radiation; Fma, forceps major; Fmi, forceps minor; ILF, inferior longitudinal fasciculus; L, left; R, right; FA, fractional anisotropy; MD, mean diffusivity.

### Mean diffusivity values

Cross-group differences of MD values are shown in Table 4. MD values were significantly different among the three groups in most of the WM tracts except for bilateral CST, IFO, and tSLF. There were significant deficits to the CgC, CgH, Fma, Fmi, left ILF, SLF, and UF in the a-MCI group compared with the NC group, while a subset of tracts including the ATR, CgC, CgH, Fma, Fmi, ILF, SLF, and UF in the CSVD-MCI group had significant deterioration from the NC group. Additionally, a statistically significant difference between the a-MCI and CSVD-MCI subjects was only present in the MD values of Fma and Fmi, as shown in Table 3 and Figure 1.

**TABLE 4:** Comparisons of mean diffusivity (MD) values.

Tracts	NC (*n* = 20)	a-MCI (*n* = 33)	CSVD-MCI (*n* = 25)	*F*-value	*P*-value
ATR.L	0.105 ± 0.022	0.109 ± 0.024	0.127 ± 0.027	3.785	0.027 ^b^
ATR.R	0.101 ± 0.022	0.108 ± 0.025	0.125 ± 0.026	3.542	0.034^b^
CST.L	0.085 ± 0.005	0.086 ± 0.006	0.088 ± 0.007	1.531	0.223
CST.R	0.086 ± 0.005	0.086 ± 0.006	0.089 ± 0.007	1.047	0.356
CgC.L	0.079 ± 0.011	0.086 ± 0.006	0.085 ± 0.005	6.961	0.002^ab^
CgC.R	0.079 ± 0.007	0.086 ± 0.005	0.085 ± 0.007	6.145	0.003^ab^
CgH.L	0.084 ± 0.013	0.106 ± 0.028	0.100 ± 0.008	5.773	0.005^ab^
CgH.R	0.085 ± 0.012	0.106 ± 0.020	0.100 ± 0.014	8.148	0.001^ab^
Fma	0.092 ± 0.010	0.100 ± 0.008	0.109 ± 0.011	13.897	<0.001^abc^
Fmi	0.085 ± 0.020	0.096 ± 0.008	0.106 ± 0.010	11.298	<0.001^abc^
IFO.L	0.089 ± 0.007	0.092 ± 0.007	0.095 ± 0.007	2.131	0.126
IFO.R	0.087 ± 0.005	0.091 ± 0.007	0.094 ± 0.007	2.343	0.103
ILF.L	0.082 ± 0.008	0.087 ± 0.005	0.089 ± 0.005	4.566	0.014^ab^
ILF.R	0.084 ± 0.009	0.086 ± 0.006	0.091 ± 0.006	4.495	0.014^b^
SLF.L	0.085 ± 0.008	0.091 ± 0.007	0.094 ± 0.007	5.898	0.004^ab^
SLF.R	0.084 ± 0.009	0.092 ± 0.010	0.095 ± 0.008	5.848	0.004^ab^
UF.L	0.088 ± 0.011	0.096 ± 0.009	0.099 ± 0.014	4.418	0.015^ab^
UF.R	0.088 ± 0.010	0.096 ± 0.008	0.099 ± 0.012	5.909	0.004^ab^
tSLF.L	0.079 ± 0.006	0.083 ± 0.004	0.083 ± 0.006	2.998	0.056
tSLF.R	0.079 ± 0.006	0.081 ± 0.004	0.083 ± 0.006	2.290	0.109

MD values are (mean ± SD) × 10^–2^ mm^2^ s^–1^. NC, normal controls; a-MCI, amnestic mild cognitive impairment; CSVD-MCI, mild cognitive impairment resulting from cerebral small vessel disease; ATR, anterior thalamic radiation; CST, corticospinal tract; CgC, cingulum (cingulate gyrus); CgH, cingulum (hippocampus); Fma, forceps major; Fmi, forceps minor; IFO, inferior fronto-occipital fasciculus; ILF, inferior longitudinal fasciculus; SLF, superior longitudinal fasciculus; UF, uncinate fasciculus; tSLF, superior longitudinal fasciculus-temporal part; L, left; R, right; MD, mean diffusivity.

*Post-hoc* paired comparisons showed significant group differences: ^a^*P* < 0.05 between NC and a-MCI; ^b^*P* < 0.05 between NC and CSVD-MCI; ^c^*P* < 0.05 between a-MCI and CSVD-MCI.

### Discriminative power of major indicators between the mild cognitive impairment subgroups

In significant pairwise comparisons, only the SCWT C performance time, the SCWT C errors, the FA values of bilateral ATR, Fmi, right ILF, and the MD values of Fma and Fmi were significantly different between the two MCI groups. The above covariates and age, gender, and years of education were entered into a binary logistic regression model, using forward: LR approach. Finally only the FA value of right ILF (*P* < 0.05, OR = 0.737, 95% CI: 0.569–0.954) and the MD value of Fmi (*P* < 0.05, OR = 2.710, 95% CI: 1.210–6.071) were statistically significant (Table 5). The diagnostic accuracy of these major indices that showed significant differences between a-MCI and CSVD-MCI were explored by ROC analysis. As results shown in Table 6, the SCWT color performance time had no diagnostic significance while the SCWT color errors had moderate discriminating potential (AUC = 0.747; sensitivity = 76.0%; specificity = 63.6%; *P* = 0.001; 95% CI: 0.617–0.877). Six diffusion tensor parameters also had differential diagnostic significance, then the MD value of Fmi showed the best discriminating power (AUC = 0.815; sensitivity = 88.0%; specificity = 72.7%; *P* < 0.001; 95% CI: 0.698–0.932).

**TABLE 5:** Binary logistic regression for significant variables between the mild cognitive impairment (MCI) groups.

Variables	B	S.E.	Wald	*P*-value	OR	95% CI
ILF.R.FA	–0.306	0.132	5.361	0.021	0.737	0.569–0.954
Fmi.MD	0.997	0.412	5.867	0.015	2.710	1.210–6.071

FA values × 10^–2^mm^2^s^–1^; MD values × 10^–4^mm^2^s^–1^. ILF, inferior longitudinal fasciculus; Fmi, forceps minor; R, right; FA, fractional anisotropy; MD, mean diffusivity; CI, confidence interval.

**TABLE 6:** Receiver operating characteristic (ROC) analysis of major indices between amnestic mild cognitive impairment (a-MCI) and cerebral small vessel disease-mild cognitive impairment (CSVD-MCI).

Index	Sensitivity	Specificity	AUC	*P*-value	95% CI
SCWT C (s)	–	–	0.551	0.510	0.400–0.702
SCWT C errors	76.0%	63.6%	0.747	0.001	0.617–0.877
ATR.L.FA	80.0%	66.7%	0.765	0.001	0.641–0.889
ATR.R.FA	80.0%	72.7%	0.766	0.001	0.640–0.892
Fmi.FA	88.0%	60.6%	0.739	0.002	0.611–0.868
ILF.R.FA	96.0%	54.5%	0.776	<0.001	0.657–0.894
Fma.MD	68.0%	75.8%	0.718	0.005	0.582–0.854
Fmi.MD	88.0%	72.7%	0.815	<0.001	0.698–0.932

AUC, area under curve; SCWT, Stroop color word test; ATR, anterior thalamic radiation; Fma, forceps major; Fmi, forceps minor; ILF, inferior longitudinal fasciculus; L, left; R, right; FA, fractional anisotropy; MD, mean diffusivity; CI, confidence interval.

## Discussion

Previous studies revealed episodic memory impairment in AD patients and impaired attention and executive function in CSVD patients (Rosas et al., 2021; Zanon Zotin et al., 2021), and suggested a non-specific neuropsychological profile for CSVD-MCI and a more specific cognitive pattern in MCI due to AD (Zhou and Jia, 2009). However, the results of the cognitive impairment profile in different etiologies of MCI patients were discordant since some authors found deficient episodic memory and speed/attention in MCI due to AD (Nordlund et al., 2008) while other study reported memory and executive function were mainly impaired in MCI patients of AD origin and multiple cognitive domains were impaired in CSVD-MCI group (Zhou and Jia, 2009). We administered comprehensive neuropsychological tests to find that compared with controls, a-MCI patients had worse performance only in the cognitive domains of memory and language functions. The current study revealed extensive cognitive deficits in four domains including memory, language, attention, and executive functions among patients with CSVD-MCI. Moreover, the two patient groups differed mainly on measures of the Stroop test, indicating that working memory and executive function were more dysfunctional in CSVD-MCI patients. Executive dysfunction is considered to be “the core characteristic of vascular cognitive impairment” (Roman et al., 2004). Studies have shown that executive function is the most sensitive cognitive domain in deterioration of the microstructural integrity of WM of vascular origin (O’Sullivan et al., 2004; Nitkunan et al., 2008).

Nordlund et al. (2011) found that the results of other neuropsychological tests in MCI resulting from AD and vascular dementia were basically consistent except for extremely poor performance of MCI patients of AD origin on memory tests. Other researchers reported differences in processing speed, memory, and visuospatial skills between the two different MCI patients (Luis et al., 2004; Zhou and Jia, 2009). Nevertheless, there was no significant difference in memory function between the two groups in the current study. These findings were inconsistent with the findings of our study and may be due to differences in subjects. Vascular MCI included non-cavitary cortical or subcortical infarction, infarction in key areas such as thalamus or basal ganglia, or large vessel hemorrhage and signs of infarction. However, our CSVD-MCI group in this study explicitly excluded patients with these conditions.

Furthermore, we found the deterioration of CgC, CgH, Fma, Fmi, left IFO, ILF, SLF, and UF in a-MCI subjects. The degeneration of WM tracts in a-MCI patients in this study were extensive and spread throughout the frontal, temporal, parietal, and other regions. Previous studies have shown that mainly damaged WM tracts including ATR, Fma, Fmi, cingulate tract, para-hippocampal cingulate tract, and IFO in early AD connected the earliest affected gray matter structures (i.e., hippocampus, cingulate gyrus, medial prefrontal cortex, and posterior cingulate cortex) in AD patients (Villain et al., 2010; Luo et al., 2019), basically consistent with the conclusion of our study.

Additionally, we observed the ATR, CgC, CgH, Fma, Fmi, IFO, ILF, SLF, and UF are impaired in patients with CSVD-MCI. The WM microstructure damage in the CSVD-MCI group was more extensive and severe than the a-MCI group, which may be related to the CSVD-MCI subjects included in this study having high visual scores of WMLs. Previous studies have found that the damaged WM in CSVD-MCI is mainly located in the prefrontal pathway of the thalamus and caudate lobe (e.g., ATR, Fmi, IFO, ILF, SLF) which are significantly associated with the executive and attention functions (Mamah et al., 2010; Biesbroek et al., 2017). As part of the anterior striatal thalamic circuit, ATR have an important effect on executive function (Mamah et al., 2010). In our study, significant impairment to ATR was not observed in the a-MCI group, meanwhile executive function was relatively preserved.

This study found that damaged tracts in a-MCI were mostly involved in memory function, while damaged tracts in CSVD-MCI were mostly associated with executive function. By comparing the two MCI groups directly, there were significant differences in the DTI parameters of bilateral ATR, Fma, Fmi, and right ILF. The degeneration of the above tracts in the CSVD-MCI group were more severe than the a-MCI group, which may be explained by more participation of these WM tracts in executive function. The Fma mainly helps the communication and connection between the hemispheres, and the integrity of WM microstructure is related to visuospatial function and working memory (Krogsrud et al., 2018). Fmi is connected to the bilateral prefrontal cortex, which is of the substantive circuits connecting the bilateral regions of the default mode network playing an important role in executive function (Fox et al., 2005; van den Heuvel et al., 2009). ILF is the main connecting fiber tract of the frontal, parietal, temporal, and occipital cortex involved in executive function and processing speed (Biesbroek et al., 2017). Binary logistic regression and ROC analysis in this study further confirmed that the altered right ILF and Fmi are important WM tracts for predicting the two MCI subtypes.

This study found significant differences in DTI parameters of the corpus callosum (including the Fma and Fmi) between each MCI subtype. ROC analyses showed that the MD value of Fmi was the best index to distinguish between the two MCI groups, showing the importance of corpus callosum. Previous researchers have suggested that corpus callosum is the most important structure to discriminate different types of dementia (Zarei et al., 2009). The WM microstructure of corpus callosum, especially the Fmi, showed significant difference in vascular dementia and AD patients (Zarei et al., 2009). Palesi et al. (2018) found that the FA value of corpus callosum decreased and all DTI parameters of corpus callosum changed in vascular dementia patients, suggesting that the damage of corpus callosum was much more severe than AD patients.

Importantly, only two neuropsychological tests and six diffusion parameters varied significantly between the two MCI groups. The Stroop test did not differ significantly in the binary logistic regression model, and based on the above results of ROC analysis, the diagnostic efficacy of the neuropsychological tests were not as high as that for the diffusion parameters, suggesting that neuroimaging markers might be more reliable than neuropsychological tests in discriminating a-MCI from CSVD-MCI. Application of neuroimaging, especially DTI, is more conducive to identifying the etiology of MCI than neuropsychological tests before more sensitive diagnostic tools are applied to the clinical and practice. Further work is needed to verify the diagnostic efficacy of DTI indices in larger samples in the future.

In conclusion, comparing cognitive deficits and the deterioration of WM tracts in the two MCI groups highlights the significance of etiological diagnosis of MCI. The MD value of Fmi could serve as an optimal indicator to differentiate between a-MCI and CSVD-MCI. Quantitative metrics of DTI should been recommended as part of the diagnostic pipeline for the etiological diagnosis of MCI.

## Limitations

There are still some limitations in this study. First, the study was of a cross-sectional design without follow-up; the diagnosis of MCI lacked pathological evidences and whether the MCI patients would progress to AD or CSVD in the future was uncertain. Second, our study was a single-center study with a relatively small sample size, so there might be a certain degree of selection bias. In order to improve the reliability of the research results, a multi-center study with a larger sample size is needed in the future. Finally, due to the machine limitation, the diffusion gradient direction of MRI can only be set at 30.

## Data availability statement

The original contributions presented in this study are included in the article/supplementary material, further inquiries can be directed to the corresponding authors.

## Ethics statement

The studies involving human participants were reviewed and approved by the Institutional Review Board of the First Affiliated Hospital of Soochow University. The patients/participants provided their written informed consent to participate in this study.

## Author contributions

YW and HD designed this study. YZ finished the statistical analysis and submitted the manuscript. YZ, MF, LD, HS, and ZZ collected the participants’ data and wrote the manuscript. YZ, LL, and YQ revised the manuscript. All authors gave final agreement to be accountable for all aspects of the work in ensuring that questions related to the accuracy or integrity of any part of the work were appropriately investigated and resolved, contributed to the article, and approved the submitted version.
